# Editorial: Applications of Genome Sequences for Discovering Characteristics that Are Unique to Different Groups of Organisms and Provide Insights into Evolutionary Relationships

**DOI:** 10.3389/fgene.2016.00027

**Published:** 2016-02-19

**Authors:** Radhey S. Gupta

**Affiliations:** Department of Biochemistry and Biomedical Sciences, McMaster UniversityHamilton, ON, Canada

**Keywords:** comparative genomics, microbial classification, molecular signatures, genetic and biochemical characteristics, conserved signature indels

Genome sequences are making available an unprecedented amount of genetic information that has the potential to reliably elucidate many aspects of physiology, biochemistry, and evolutionary relationships of different organisms. For efficient analyses of the vast amount of genomic sequence data, new reductive approaches are needed which can identify reliable genetic characteristics that are specific for either particular species or related groups of organisms. These characteristics provide novel means for distinguishing different organisms and for understanding their evolutionary history as well as novel tools for phenotypic, behavioral, and biochemical studies. The papers collected in this research topic discuss the application of comparative genomic techniques in a wide range of modalities covering the evolution, classification, identification, and characterization of bacteria and viruses.

The evolutionary relationships of bacterial organisms form the underlying basis of their classification. Koton et al. utilize genomic sequence data to elucidate the evolutionary interrelationships of *Vibrio vulnificus* strains and to both trace the origin and identify unique genomic characteristics of *V. vulnificus* biotype 3. The comparative genomic analyses completed by Koton et al. are a model of how to utilize the comparative analysis of core, accessory, and unique genomic elements in order to understand the interrelationships of various strains of a single bacterial species. Bacterial classification at the genus and species level, however, is highly structured and often based on the characterization of a limited number of strains (often a single isolate) focussed on the 16S rRNA gene and a number of phenotypic and chemotaxonomic properties (Tindall et al., 2010). Sutcliffe argues that the current standards for classification have limited utility, are time consuming to perform, and are inadequate to face the exponentially growing number bacterial isolates being discovered. He suggests that future classification should focus on utilizing genome sequence data and genome derived characteristics in order to define and delineate bacterial groups. Sawana et al. provide a practical example of this type of genome-centric classification. In their study they utilize genome sequence data to identify multiple highly specific molecular signatures or characteristics which differentiate two main groups within the large and diverse genus *Burkholderia*. They then divide the genus *Burkholderia* into two genera based on these genome-derived characteristics (viz. *Burkholderia* and *Paraburkholderia*). This group of researchers has previously applied similar techniques to divide the genus *Borrelia* into two genera, *Borrelia* and *Borreliella* (Adeolu and Gupta, 2014), and the genus *Thermotoga* into two genera, *Thermotoga* and *Pseudothermotoga* (Bhandari and Gupta, 2014). The benefits of basing classification on genome sequence analysis are numerous. Firstly, genome sequence data is more easily and consistently comparable between different organisms than phenotypic or chemotaxonomic properties which allows for more reliable and consistent delineation between organisms. Secondly, genetic and molecular characteristics universally shared by a defined group of organisms are more stable over time than chemotaxonomic characteristics and are unique to the group for which they were identified facilitating their use in the identification and classification of novel members of the group. Lastly, the identified genomic and molecular differences form the basis of any recognized or unrecognized phenotypic or biochemical differences between the different groups of organisms, thus, they are important novel targets of study and should lead to a greater focus on understanding of the underlying biological differences between the indicated groups. The utility of comparative genomic analysis in studying the underlying biological differences between groups of organisms is exemplified by Yang et al. who utilize the comparative analysis of *Microcystis aeruginosa* genomes to identify the CRISPR-Cas systems of different *Microcystis aeruginosa* subgroups, giving novel insights into their resistance to bacteriophages and their ability to modulate genomic stability and plasticity. Liu et al. utilize a number of biochemical assays to characterize the function of a laterally transferred gene cluster containing the *tetR* gene, identified via comparative genomic techniques. They show that the *tetR* gene and TetR protein contribute to cell survival under oxidative stress and are able to identify a number of regulatory roles played by the TetR protein. Taken together, these two studies exemplify the utility of studying and characterizing the genome derived characteristics that are beginning to be used to classify bacterial groups.

Viruses and virus-like particles represent the most abundant sources of DNA in the environment (Weinbauer, 2004). Due to the size, plasticity, and unique composition of viral genomes, they represent particularly interesting candidates for comparative analysis. Despite this, viral genome are relatively unexplored in comparison to both eukaryotic and prokaryotic genomes. Chan et al. use genome sequence data to reveal the evolutionary relationships between the members of N4-like phage genus and to define the core genome of all members of the N4-like phage genus and the core genome of the N4-like *Roseobacter* phages, providing novel insight into the traits which characterize the genus and their evolutionary relationships. Mohiuddin and Schellhorn utilize environmental sampling and comparative genomic analyses of environmental DNA and DNA extracted from virus-like particles to examine the abundance and distribution of different classes of viruses in freshwater environments. These studies both represent useful applications of genomic sequence data and genome derived characteristics to identify and characterize viral groups and populations that are currently underexplored.

The papers presented in this research topic exemplify the broad range of applications of comparative genomic analysis and genome derived characteristics in biology and related sciences. The use of genome sequence data to answer novel questions or challenge previously held assumptions is revolutionizing all fields of biology and novel methods and applications of comparative genomics are becoming an indispensable part of modern biological research.

## Author contributions

RG was the Editor for this Research Topic. I have critically read all of the papers submitted under this research topic and have written/finalized the submitted Editorial.

### Conflict of interest statement

The author declares that the research was conducted in the absence of any commercial or financial relationships that could be construed as a potential conflict of interest.
